# Comparison of programs for determining temporal-spatial gait variables from instrumented walkway data: PKmas versus GAITRite

**DOI:** 10.1186/1756-0500-7-542

**Published:** 2014-08-18

**Authors:** Thorlene Egerton, Pernille Thingstad, Jorunn L Helbostad

**Affiliations:** Department of Neuroscience, Norwegian University of Science and Technology, Trondheim, Norway; Department of Clinical Services, St. Olav University Hospital, Trondheim, Norway

**Keywords:** Gait, GAITRite, PKMAS, Reliability, Aging

## Abstract

**Background:**

Measurement of temporal-spatial gait variables is common in aging research with several methods available. This study investigated the differences in temporal-spatial gait outcomes derived from two different programs for processing instrumented walkway data.

**Method:**

Data were collected with GAITRite® hardware from 86 healthy older people and 44 older people four months following surgical repair of hip fracture. Temporal-spatial variables were derived using both GAITRite® and PKmas® processing programs from the same raw footfall data.

**Results:**

The mean differences between the two programs for most variables were negligible, including for Speed (mean difference 0.3 ± 0.6 cm/sec, or 0.3% of the mean GAITRite® Speed). The mean absolute percentage difference for all 18 gait variables examined ranged from 0.04% for Stride Duration to 66% for Foot Angle. The ICCs were almost perfect (≥0.99) for all variables apart from Base Width, Foot Angle, Stride Length Variability, Step Length Variability, Step Duration Variability and Step Width Variability, which were all never-the-less above 0.84. There were systematic differences for Base Width (PKmas® values 1.6 cm lower than GAITRite®) and Foot Angle (PKMAS® values 0.7° higher than GAITRite®). The differences can be explained by the differences in definitions and calculations between the programs.

**Conclusions:**

The study demonstrated that for most variables the outcomes from both programs can be used interchangeably for evaluation of gait among older people collected with GAITRite® hardware. However, validity and reliability for Base Width and Foot Angle derived by PKMAS® would benefit from further investigation.

**Electronic supplementary material:**

The online version of this article (doi:10.1186/1756-0500-7-542) contains supplementary material, which is available to authorized users.

## Background

Gait analysis provides highly relevant outcomes for the older population. It reflects both impairment-level deficits and functional status [1–3]. Temporal-spatial gait variables have repeatedly been shown to be important for identification of injury/disease [4–6], prediction of falls [7, 8], and quantification of the effect of interventions [9, 10]. In particular, gait speed has been associated with health status, activity levels and quality of life, and is predictive of future morbidity and mortality [11–14].

The GAITRite® system is a well established method of quantifying gait. Over 200 papers have been published since 2000 using data collected and processed with the GAITRite® system. The measurement properties of a large number of temporal and spatial outcomes derived from GAITRite® data have been reported (eg. [15–17]). Recently, a new program has been developed in order to solve some of the problems with processing difficult footstep patterns, for example overlapping steps and turns. The PKmas® software purports to accurately derive temporal-spatial outcomes from raw GAITRite® data. However, in order to interpret clinical and research findings from PKmas® processed gait data, and to be able to draw comparisons with published data that has used the GAITRite® system, the inter-program reliability of the two processing algorithms needed to be examined. A direct comparison of outcomes from the same walk trials would enable the degree of variability caused by the processing program alone to be determined, irrespective of other sources of noise in the data.

This study examined the level of agreement and inter-program variability between the two processing programs, using data from older people walking at self-selected, preferred speed, on a GAITRite® mat. Very high levels of agreement for an outcome variable would indicate the variable is interchangeable regardless of the program used to process it. Systematic differences, if known, can be taken into consideration during comparisons. Lower levels of agreement due to random spread of differences would suggest the outcome may have important differences when processed with PKmas®, and the reliability and validity of the variable should not be assumed to be the same as with GAITRite®.

## Methods

### Participants

Data from two groups of participants were used for this study. The first group consisted of 100 healthy older people from the community in Trondheim, Norway. They were recruited for the Generation 100 study, an exercise intervention study (ClinicalTrials.gov identifier: NCT01666340). The second group included 50 older people, who were tested four months after surgical repair of hip fracture. The hip fracture patients were all part of the Trondheim Hip Fracture Trial [18]. All participants gave written informed consent to participate in their respective studies. Ethical approvals for the studies, which included the use of their data for purposes of cross-sectional and methods analyses, were granted by the Norwegian Ethical Review Board for Medical and Health Research (REK) – South East Region (2013/787b) and the Regional Committee of Ethics in Medical Research (Mid-Norway) (REK4.2008.335) respectively.

### Procedures

For the healthy group, the baseline GAITRite® (CIR Systems Inc, Havertown, PA) raw data was collected using a 5.5 m mat (active length). Participants were asked to walk along the walkway at their preferred (usual) speed starting and stopping at least 1 m outside the ends of the mat (total walkway length at least 8.7 m). The hip fracture group were similarly asked to walk along a 4.7 m GAITRite® mat (total walkway at least 7.7 m) at their preferred speed. Only the first pass was used for this study.

The raw data was processed with both GAITRite® (v3.8E) and PKmas® (v507C4i3) (ProtoKinetics, Havertown, PA) software and exported to Excel. After processing, all walks were checked to ensure the same steps, as well as the same number of steps, were used in both processing methods. Thirteen healthy participants and six hip fracture participants were excluded because during the processing of the walk files, a different number of steps were retained. A slight variation in which footfalls are retained would lead to small differences in the outcome variable values. This difference is likely to be clinically insignificant, but we wanted to exclude all sources of variation apart from those caused by the different software algorithms. It was noted that when the walk had two or fewer footfalls with one foot, PKmas® does not calculate standard deviation (SD) for ipsilateral Stride Length, Step Length, Stride Duration, Step Duration and Base Width. In GAITRite®, SD of Stride Length, Stride Duration and Base Width are not calculated. When there is no SD calculated, PKmas® exports a blank cell to Excel, however GAITRite® exports a zero. This creates an error when the right and left values are averaged. For this reason we excluded walks where there were less than six footfalls in total. One healthy participant was excluded for this reason.

### Outcome variables

There are many gait variables that can be derived from data collected with GAITRite® mats. The outcome variables compared in this study were chosen as those previously reported in validity and/or reliability studies using the GAITRite® system (eg. [15–17], further information is provided in Additional file 1: A). The included variables were those that are calculated from the footfalls themselves, rather than variables that are derived from other gait variables. Thus symmetry variables and composite scores were not examined. Exceptions to this are Speed which is combines Stride Length and Stride Duration, and the ‘percentage of gait cycle’ variables. For all variables apart from Speed and Cadence, the mean of the left and right values were calculated and used as a single data point for the variable.

### Statistical analyses

Mean difference between values for each outcome variable from the two programs, and the percentage error (mean of the *absolute* difference expressed as a proportion of the GAITRite® value) were obtained for each group to identify the magnitude of the differences between the processing algorithms. The mean percentage difference underestimates the variability at individual level if differences are both positive and negative. The mean *absolute* percentage differences were therefore calculated to better indicate the size of the error at individual level. The mean differences for the total cohort are also presented with this difference expressed as a percentage of the mean GAITRite® value. Intraclass correlation coefficients (ICC) for absolute agreement (2,1) and consistency (3,1) were calculated for each pair of outcomes to determine inter-program reliability [19]. Absolute agreement indicates how close individual data points are to each other using the two programs, while consistency indicates the relative agreement or agreement regardless of systematic error [20]. The Bland-Altman method was used to calculate the 95% limits of agreement (LOA) to demonstrate the spread of differences [21], and mean versus difference plots were inspected in order to identify heteroscedasticity in the differences over the range of values.

## Results

The final cohort consisted of 86 healthy and 44 hip fracture participants who had mean age ± SD of 72.0 ± 1.3 years and 82.7 ± 6.0 years respectively. Fifty-six percent of the healthy group and 82% of the hip fracture group were women. Table 1 presents the group means for each group, each program and each variable, plus the mean difference between the values generated by each processing program and mean absolute percentage differences. The mean differences between programs were similar for both groups of participants, although the mean absolute percentage difference was sometimes higher among the healthy group for the variability measures because the SD values tended to be lower among the healthier older people.

**Table 1:** **Data for each outcome variable**

	***Healthy group***			***Hip fracture group***		
	GAITRite® (mean ± SD)	PK**MAS®** (mean ± SD)	Mean difference* ± SD (% error)	GAITRite® (mean ± SD)	PK**MAS®** (mean ± SD)	Mean difference* ± SD (% error)
Speed (cm/s)	129 ± 21	129 ± 21	0.3 ± 0.6 (0.4%)	60 ± 22	61 ± 23	0.4 ± 0.5 (0.9%)
Cadence (steps/min)	110 ± 10	110 ± 10	−0.1 ± 0.2 (0.1%)	93 ± 15	92 ± 15	−0.0 ± 0.1 (0.1%)
Stride length (cm)	140 ± 16	140 ± 16	−0.0 ± 0.2 (0.1%)	78 ± 25	78 ± 25	0.1 ± 0.6 (0.3%)
Step length (cm)	70 ± 8	70 ± 8	−0.1 ± 0.4 (0.5%)	39 ± 13	39 ± 13	0.2 ± 0.3 (0.7%)
Stride duration (s)	1.1 ± 0.1	1.1 ± 0.1	0.00 ± 0.00 (0.04%)	1.3 ± 0.2	1.3 ± 0.2	0.00 ± 0.00 (0.1%)
Step duration (s)	0.55 ± 0.05	0.55 ± 0.05	0.000 ± 0.003 (0.5%)	0.67 ± 0.11	0.67 ± 0.11	−0.001 ± 0.004 (0.5%)
Stance duration (s)	0.69 ± 0.07	0.69 ± 0.07	0.003 ± 0.006 (1.2%)	0.93 ± 0.18	0.94 ± 0.18	0.011 ± 0.019 (1.4%)
Swing duration (s)	0.41 ± 0.03	0.41 ± 0.03	−0.002 ± 0.004 (0.5%)	0.40 ± 0.08	0.40 ± 0.08	−0.004 ± 0.007 (1.3%)
Double support duration (s)	0.28 ± 0.04	0.28 ± 0.05	0.004 ± 0.008 (1.5%)	0.53 ± 0.16	0.53 ± 0.16	0.009 0.013 (1.8%)
Stance time as a percentage of cycle time (%)	62.6 ± 1.3	62.8 ± 1.4	0.17 ± 0.35 (0.3%)	69.6 ± 4.5	69.9 ± 4.5	0.30 ± 0.46 (0.5%)
Double support time as a percentage of cycle time (%)	25.3 ± 2.6	25.7 ± 2.8	0.39 ± 0.84 (2.1%)	39.3 ± 8.9	39.8 ± 9.1	0.52 ± 0.98 (1.9%)
Base width (cm)	8.7 ± 2.5	7.1 ± 2.8	−1.64 ± 0.71 (21.4%)	10.4 ± 3.7	8.9 ± 3.9	−1.58 ± 1.00 (19%)
Foot angle (°)	6.8 ± 3.8	7.5 ± 3.7	0.65 ± 1.02 (66%)	7.7 ± 5.7	8.5 ± 5.6	0.76 ± 0.82 (40%)
Variability (SD) in Stride Length (cm)	2.4 ± 1.2	2.6 ± 1.2	0.17 ± 0.50 (28%)	4.1 ± 1.9	4.1 ± 1.8	0.00 ± 0.43 (9%)
Variability (SD) in Step Length (cm)	1.6 ± 0.7	1.7 ± 0.8	0.03 ± 0.55 (32%)	2.7 ± 1.1	2.7 ± 1.0	−0.08 ± 0.56 (17%)
Variability (SD) in Stride Duration (s)	0.02 ± 0.01	0.02 ± 0.01	0.001 ± 0.003 (7%)	0.07 ± 0.04	0.07 ± 0.04	0.000 ± 0.002 (2.3%)
Variability (SD) in Step Duration (s)	0.01 ± 0.01	0.01 ± 0.01	−0.001 ± 0.005 (20%)	0.04 ± 0.02	0.04 ± 0.02	−0.001 ± 0.004 (8%)
Variability (SD) in Step Width (cm)	1.9 ± 0.9	2.0 ± 0.9	0.05 ± 0.18 (9%)	1.8 ± 0.8	2.0 ± 0.9	0.12 ± 0.23 (11%)

*Negative differences indicate GAITRite® higher than PKmas®.

SD = standard deviation.

Mean ± SD, mean difference ± SD and mean absolute percentage error, for each group, each system and each variable.

Table 2 presents the results of the ICCs, differences for the total cohort, and LOA. The inter-program reliability was very high (both ICCs ≥ 0.99, p < 0.001) for Speed, Cadence, Stride Length, Step Length, Stride Duration, Step Duration, Stance Duration, Swing Duration, Double Support Duration, Stance%, Double Support% and Stride Duration Variability. ICC(2,1) showed absolute agreement above 0.95 for all others except Base Width (0.86) and Step Length Variability (0.84). ICC(3,1) was similar to absolute agreement for all measures except Base Width where consistency was very high at 0.97. High consistency but lower absolute agreement indicates that there was a systematic difference in the Base Width values.

**Table 2:** **Intraclass correlations and limits of agreement**

Gait variable	Absolute agreement: ICC(2,1) (95% CI)	Consistency: ICC(3,1) (95% CI)	Mean difference* (SD,% difference)	Limits of agreement 95% CI	
				Lower	Upper
Speed (cm/s)	1.00 (1.00-1.00)	1.00 (1.00-1.00)	0.34 (0.59, 0.3%)	−0.82	1.50
Cadence (steps/min)	1.00 (1.00-1.00)	1.00 (1.00-1.00)	−0.05 (0.19, 0.0%)	−0.42	0.33
Stride length (cm)	1.00 (1.00-1.00)	1.00 (1.00-1.00)	0.02 (0.38, 0.0%)	−0.73	0.76
Step length (cm)	1.00 (1.00-1.00)	1.00 (1.00-1.00)	0.02 (0.42, 0.0%)	−0.79	0.84
Stride duration (s)	1.00 (1.00-1.00)	1.00 (1.00-1.00)	0.000 (0.001, 0.0%)	−0.002	0.003
Step duration (s)	1.00 (1.00-1.00)	1.00 (1.00-1.00)	0.000 (0.004, −0.1%)	−0.008	0.007
Stance duration (s)	1.00 (1.00-1.00)	1.00 (1.00-1.00)	0.005 (0.009, 0.7%)	−0.012	0.022
Swing duration (s)	0.99 (0.99-1.00)	1.00 (0.99-1.00)	−0.003 (0.005, −0.7%)	−0.013	0.007
Double support duration (s)	1.00 (0.99-1.00)	1.00 (1.00-1.00)	0.005 (0.010, 1.5%)	−0.014	0.025
Stance time as a percentage of cycle time (%)	1.00 (0.99-1.00)	1.00 (0.99-1.00)	0.22 (0.39, 0.3%)	−0.56	1.00
Double support time as a percentage of cycle time (%)	0.99 (0.99-1.00)	1.00 (0.99-1.00)	0.43 (0.89, 1.4%)	−1.31	2.17
Base width (cm)	0.86 (−0.03-0.96)	0.97 (0.95-0.98)	−1.62 (0.82, −17.4%)	−3.22	−0.02
Foot angle (°)	0.97 (0.89-0.99)	0.98 (0.97-0.98)	0.69 (0.95, 9.7%)	−1.18	2.56
Variability (SD) in Stride Length (cm)	0.95 (0.93-0.97)	0.96 (0.94-0.97)	0.01 (0.48. 3.6%)	−0.84	1.06
Variability (SD) in Step Length (cm)	0.84 (0.78-0.89)	0.84 (0.78-0.89)	−0.01 (0.56, −0.5%)	−1.10	1.08
Variability (SD) in Stride Duration (s)	1.00 (0.99-1.00)	1.00 (0.99-1.00)	0.001 (0.003, 1.6%)	−0.006	0.007
Variability (SD) in Step Duration (s)	0.98 (0.97-0.98)	0.98 (0.97-0.98)	−0.001 (0.005, −2.7%)	−0.009	0.008
Variability (SD) in Step Width (cm)	0.97 (0.95-0.98)	0.98 (0.97-0.98)	0.08 (0.20, 3.9%)	−0.32	0.47

ICC = Intraclass Correlation, CI = confidence interval, SD = standard deviation.

*Negative differences indicate GAITRite® higher than PKmas®.

ICC (2,1) absolute agreement, ICC(3,1) consistency (with 95% CI), mean difference (with SD and mean difference as a percentage of the mean GAITRite® value), and 95% limits of agreement for the total cohort. All ICCs were significant at p < 0.001.

The magnitudes of the mean differences between the two programs were very small relative to the magnitudes of the variables themselves for all measures apart from Base Width (mean difference −1.6 cm, or 17.4% of mean GAITRite® value) and Foot Angle (mean difference 0.7°, or 9.7% of mean GAITRite® value). Mean absolute percentage differences showed individual differences could be quite large for all of the variability measures except Stride Duration Variability. Mean absolute percentage differences were also large for Base Width (around 20%, differences ranged from −4.1 to 0.4 cm) and Foot Angle (range −2.6 to 3.5°). The magnitude of the differences was especially high for Foot Angle with mean absolute percentage difference for the cohort of 57%.

**Figure 1:**
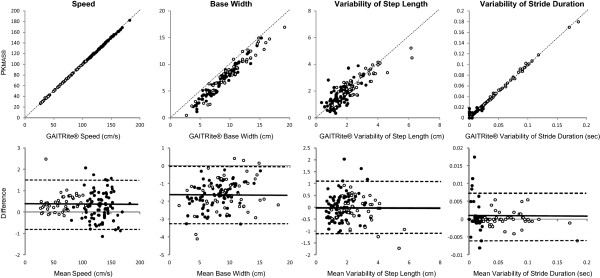
**Associations between GAITRite® and PK** **MAS** **® data.** Scatter plots showing the associations between GAITRite® and PKMAS® data, and Bland-Altman plots showing mean difference and 95% limits of agreement for Speed, Base Width, Step Length Variability and Stride Duration Variability. ● = healthy older people, ○ = post hip fracture patients.

Scatter plots and Bland-Altman plots are shown for Speed, Base Width, Step Length Variability and Stride Duration Variability in Figure 1. The plot for Base Width shows >95% of differences were negative indicating that PKmas® Base Width values were systematically lower than the GAITRite® values. The plots for Stride Duration Variability (not shown) and Step Duration Variability showed greater differences for lower values of variability which affected only a small number of healthy participants. Apart from these two variables the plots showed even spread of differences over the range of values.

## Discussion

This study demonstrated high levels of absolute agreement and consistency between the new and the established algorithms for most of the temporal and spatial gait variables we examined using electronic walkway data from healthy and gait impaired older people. All ICC values were greater than 0.84 and, with the exception of Base Width and Step Length Variability, greater than 0.95. However, the study identified several variables that should be considered with some caution at group level, and a few more that could be problematic at individual level if comparing GAITRite® to PKmas®.

### Base width

The ICC(2,1) absolute agreement for Base Width was 0.86 but the ICC(3,1) for consistency was 0.97, which suggests that while absolute agreement with GAITRite® values may be lacking, and both individual and group level comparisons not recommended, the variable processed by PKmas® may be itself reliable and as good at detecting change over time as GAITRite®. PKmas® values are approximately 1.6 cm, or about 17%, lower than GAITRite® values. The systematic and random differences between the two programs can be explained by differences in how they define and calculate Base Width (see Additional file 2: B1). In essence, an outward foot angle greater than zero degrees, will lead to the GAITRite® Base Width measure being larger than the PKmas® base width measure. The greater the amount of Foot Angle, the larger the difference between the two Base Width values. It should be noted, however, that previous studies have questioned the reliability of GAITRite® Base Width as an outcome measure. Menz et al. found the test-retest ICC using the average from three walks was only 0.49 for a group of older people [16]. This suggests the within-individual variation can be close to the between-individual variability.

### Step length variability

The lower ICCs for absolute agreement and consistency for Step Length Variability suggest that the output from the two processing methods should not be considered equivalent at individual level, and considered with caution at group level. One reason is that the magnitude of the variable itself is quite small so that even small differences between the programs can result in *relatively* large values for the differences between the values. In addition, step spatial calculations are different in the two processing methods (Additional file 2: B2). These small differences that do not noticeably affect the resulting values for Step Length if the walk is reasonably straight, can result in relatively larger differences in the SD of Step Length. If the direction of progression of the walk is not parallel to the mat, the values, and SDs of the values, can differ between the two programs even more.

### Foot angle

The ICCs indicated that Foot Angle was acceptable at group and individual level although values appeared to be consistently about 0.7° higher with PKMAS®. The upper level of the 95% limit of agreement was 2.6°. These differences could be considered unacceptably large. Values for individuals were on average 57% different which also appears unacceptably large. It is important to note here that, as with Base Width, the reliability of the Foot Angle as an outcome measure has been questioned because the variability within individuals is relatively large compared with the magnitude of the variable [16]. The difference between the programs can again be explained by the different methods of calculation (Additional file 2: B3). It is not possible from this study to say which method is more valid or reliable.

### All variability measures

The agreement for variability of both the temporal and spatial stride and step values appeared to be good at group level but there were some unacceptably high absolute differences, in particular among individuals with very low variability. This seems to be due to the resolution of the standard deviation calculation when the values are close to zero. Some small values are exported as zero by GAITRite® but as greater than zero by PKmas®. The small differences in the calculation of spatial measures of Stride and Step Length can also be explained by differences in the location of the heel reference point (Additional file 2: B1). There are also differences in the calculations of temporal measures (Additional file 2: B4).

Prior studies have determined the validity and reliability for variables derived from the GAITRite® system (Additional file 1: A). GAITRite® data has been compared with paper and ink techniques, video-based systems, in-shoe stride analysers and 3-dimensional motion analysis systems [15–17, 22, 23]. The measurement error between the PKmas® and GAITRite® algorithms was found to be smaller than errors reported in these other comparisons. The clinical meaning of the magnitude of the differences needs to be considered in the light of the purpose of the measurement. The impact of the slight differences in definitions and calculations used by PKmas® for some of the variables may affect (improve or reduce) the validity of the variable in terms of its association with disease status, function and fall risk. Such studies are recommended for future research.

We chose to take the average of the values from left and right sides, rather than the average of all the steps. For most of the variables there will be negligible difference between the mean of the left and right sides and the mean of all the footfalls. However, for the variability measures, this decision is clinically important because mean SD is a better indication of the within-individual variability than the SD of all steps which will also be related to the degree of asymmetry [24]. There were also practical reasons for this approach as GAITRite® only exports left and right means and not the mean of all the footfalls. To derive the mean of all the footfalls, the individual footfalls would need to be exported. PKMAS® exports right, left and grand means. Other considerations regarding the two programs include:

1. We found that PKmas® can indeed process difficult walks that include overlapping, double or backward steps more easily than GAITRite®.
2. GAITRite® exports a zero when a value cannot be calculated, for example due to insufficient steps. This affects the SD of many variables when there are five or fewer footfalls. While only one of our healthy participants needed only five steps to cover the active walkway (5.5 m), our participants were all over 70 years and walking at preferred speed. Researchers interested in the standard deviation of walks from younger participants or people walking at faster speeds should use caution with the data exported from GAITRite®, especially with shorter mats. We also found that SD values close to zero are exported as zero by GAITRite® but as a small value by PKmas®.
3. PKmas® purports to be able to process data recorded with GAITRite® hardware, however we encountered a few problems. In particular, PKmas® periodically reads a single active sensor as a footfall and careful checking is required to identify these ‘extra’ footfalls. In addition, PKmas® occasionally had difficulty determining the duration of stance phase for the final step. This may be because both our mats have ‘seen a lot of action’, but we recommend careful checking of each walk during processing of GAITRite® data with PKmas®.

This study did not directly investigate the reliability or validity of PKmas® derived data, however for the variables with good absolute agreement and consistency and minor differences from GAITRite® derived variables, validity and reliability can be assumed to be the same as for GAITRite®. For the remaining variables, it is not possible to know from this study whether validity and reliability are better or worse than for the GAITRite® derived variables. The study aimed to directly compare the two programs and a strength of the study is that the same footsteps were used by both processing algorithms and therefore the differences found can only be explained by the processing. We included participants with a range of gait ability (preferred gait speed ranged between 27-182 cm/s) and included participants with and without gait impairment. In addition, the study used testing procedures typical of those used in research studies with this population. However, the findings cannot be generalised to all populations and testing procedures.

## Conclusions

GAITRite® is a widely used clinical and research tool and this report is an important step in determining the utility of PKmas® as an alternative processing method. We conclude that Speed, Cadence, Stride Length, Step Length, Stride Duration, Step Duration, Stance Duration, Swing Duration, Double Support Duration, Stance%, Double Support% and Stride Duration Variability values are interchangeable with GAITRite® values. Base Width and Foot Angle have systematic differences of 1.6 cm lower with PKmas® and 0.7° higher with PKmas® respectively. The relatively large, randomly spread differences found for Base Width, Foot Angle, and variability of Stride Length, Step Length, Step Duration and Step Width mean that we recommend values are not comparable at individual level. The findings from this study will help inform clinicians and researchers wishing to interpret data processed using PKmas®, and compare individual or group level data with published data that was processed using GAITRite®.

## Electronic supplementary material

Additional file 1: **A.pdf – Terminologies and definitions.** (PDF 139 KB)

Additional file 2: **B.pdf – Differences in calculations to derive variables between GAITRite® and PK** **mas®** **.** (PDF 101 KB)
