# Supplementary material for: Comparison of programs for determining temporal-spatial gait variables from instrumented walkway data: PKmas versus GAITRite
Source: BMC Res Notes. 2014 Aug 18;7:542. doi: 10.1186/1756-0500-7-542 (PMC4150969; doi:10.1186/1756-0500-7-542)
Supplement: Supplementary file 1 — Additional file 1: A.pdf – Terminologies and definitions. (PDF 139 KB) [file 13104_2013_3088_MOESM1_ESM.pdf]

Additional file A

Terminologies and definitions from both programs for the variables examined, plus examples of studies reporting reliability and/or validity of the variables derived from GAITRite® hardware and software. Information sourced from GAITRite® (http://www.gaitrite.com/downloads/WI-02-15_Technical_Reference_L.pdf) and Prokinetics (http://www.protokinetics.com/images/PKMAS_Gait_Measurement_Definitions_Short.pdf).

**Outcome variable**	**GAITRite® term and definition**	**PKMAS® term and definition**	**Studies**
Speed	Velocity (obtained after dividing the Distance Travelled by the Ambulation time)	Velocity (dividing the sum of all Stride Lengths by the sum of all Stride Times)	[1-16]
Cadence	Number of steps divided by ambulation time (sec), multiplied by 60.	Cadence (number of footfalls minus one, divided by the ambulation time)	[1-3, 5-10, 12-16]
Stride length	Stride length (distance between the heel points of two consecutive footprints of the same foot)	Stride Length (distance from the heel of one foot to the following heel of the same foot)	[1, 3, 4, 6, 8-11]
Step length	Step length (distance from the heel center of the current footprint to the heel center of the previous footprint on the opposite foot)	Step Length (distance between corresponding successive points on the heel of opposite feet measured parallel to the direction of progression for the ipsilateral stride of which it is the second part)	[1, 4, 5, 7-9, 11, 13-15, 17, 18]
Stride duration	Stride Time or Gait Cycle Time (time elapsed between the first contacts of two consecutive footfalls of the same foot)	Stride Time or Gait Cycle Time (time from first contact of one foot to the following first contact of the same foot)	[4, 6, 9-11, 19]
Step duration	Step Time (time elapsed from first contact of one foot to first contact of the opposite foot)	Step Time (period of time taken for one step and is measured from first contact of one foot to the first contact of following other foot)	[1, 2, 4, 7, 11, 13, 14, 17, 18, 20]
Stance duration	Stance Time (time elapsed between the First Contact and the Last Contact of two consecutive footfalls on the same foot)	Stance Time (time when the foot is in contact with the ground)	[2-4, 14, 17, 18]
Swing duration	Swing time (time elapsed between the Last Contact of the current footfall to the First Contact of the next footfall on the same foot)	Swing Time (period of time when the foot is not in contact with the ground)	[2, 4, 18]

Double support duration	Total Double Support (sum of Initial ad Terminal Double Support). GAITRite® gives separate values for Initial Double Support (from heel contact of one footfall to toe-off of the opposite footfall) and Terminal Double Support (from opposite footfall heel strike to support footfall toe-off).	Total Double Support (sum of Initial ad Terminal Double Support). PKmas® gives separate values for Initial Double Support Time (time when both feet are in contact with the ground at the beginning of the stance phase) and Terminal Double Support Time (time when both feet are in contact with the ground at the end of the stance phase).	[2, 4, 8, 11, 14, 20]
Stance time as a percentage of cycle time	% Stance (Stance Time presented as a percentage of the Gait Cycle Time)	Stance Percentage (Stance Time presented as a percentage of the Gait Cycle Time)	[10, 13]
Double support time as a percentage of cycle time	% Double Support (Double Support Time presented as a percentage of the Gait Cycle Time)	PKMAS® does not produce this variable. It was calculated from Initial and Terminal double support times which were expressed as a percentage of the Gait Cycle Time.	[3, 16]
Base width	H-H Base of Support or Base Width (vertical distance from heel center of one footprint to the line of progression formed by two footprints of the opposite foot)	Stride Width (distance between a line connecting the two ipsilateral foot heel contacts (the stride) and the contralateral foot heel contact between those events and is measured perpendicular to the stride)	[2, 4-6, 8-10, 18]
Foot angle	Toe In / Toe Out (angle between the line of progression and the midline of the footprint)	Toe In/Out Angle (angle between the Direction Of Progression and Foot Angle where Foot Angle is the angle of the foot's placement on the ground measured for the vector from the foot ellipse's heel to the toe)	[4, 5, 8, 17]
Variability (SD) in Stride Length		For both programs, the SD of the individual values for the variable was used, where SD = $\sqrt{\frac{\sum (x-\bar{x})^2}{(n-1)}}$ Means of the left SD and right SD were calculated.	[16]
Variability (SD) in step Length			[18]
Variability (SD) in Stride Duration			[16, 19]
Variability (SD) in Step Duration			[18]
Variability (SD) in Step Width			[18]

References

1. McDonough AL, Batavia M, Chen FC, Kwon S, Ziai J: **The validity and reliability of the GAITRite system's measurements: A preliminary evaluation**. *Arch Phys Med Rehabil* 2001, **82**(3):419-425.
2. Nelson AJ, Zwick D, Brody S, Doran C, Pulver L, Rooz G, Sadownick M, Nelson R, Rothman J: **The validity of the GaitRite and the Functional Ambulation Performance scoring system in the analysis of Parkinson gait**. *NeuroRehabilitation* 2002, **17**(3):255-262.
3. Bilney B, Morris M, Webster K: **Concurrent related validity of the GAITRite walkway system for quantification of the spatial and temporal parameters of gait**. *Gait Posture* 2003, **17**(1):68-74.

4. van Uden CJ, Besser MP: **Test-retest reliability of temporal and spatial gait characteristics measured with an instrumented walkway system (GAITRite)**. *BMC Musculoskelet Disord* 2004, **5**:13.
5. Menz HB, Latt MD, Tiedemann A, Mun San Kwan M, Lord SR: **Reliability of the GAITRite walkway system for the quantification of temporo-spatial parameters of gait in young and older people**. *Gait Posture* 2004, **20**(1):20-25.
6. Rao AK, Quinn L, Marder KS: **Reliability of spatiotemporal gait outcome measures in Huntington's disease**. *Mov Disord* 2005, **20**(8):1033-1037.
7. Webster KE, Wittwer JE, Feller JA: **Validity of the GAITRite walkway system for the measurement of averaged and individual step parameters of gait**. *Gait Posture* 2005, **22**(4):317-321.
8. Dusing SC, Thorpe DE: **A normative sample of temporal and spatial gait parameters in children using the GAITRite electronic walkway**. *Gait Posture* 2007, **25**(1):135-139.
9. Rome K, Hanchard NC: **Within-day reliability of temporal-spatial gait parameters associated with rheumatoid arthritic feet**. *Musculoskeletal Care* 2005, **3**(1):17-23.
10. Wondra VC, Pitetti KH, Beets MW: **Gait parameters in children with motor disabilities using an electronic walkway system: assessment of reliability**. *Pediatr Phys Ther* 2007, **19**(4):326-331.
11. Montero-Odasso M, Casas A, Hansen KT, Bilski P, Gutmanis I, Wells JL, Borrie MJ: **Quantitative gait analysis under dual-task in older people with mild cognitive impairment: a reliability study**. *J Neuroeng Rehabil* 2009, **6**:35.
12. Hollman JH, Childs KB, McNeil ML, Mueller AC, Quilter CM, Youdas JW: **Number of strides required for reliable measurements of pace, rhythm and variability parameters of gait during normal and dual task walking in older individuals**. *Gait Posture* 2010, **32**(1):23-28.
13. Kuys SS, Brauer SG, Ada L: **Test-retest reliability of the GAITRite system in people with stroke undergoing rehabilitation**. *Disabil Rehabil* 2011, **33**(19-20):1848-1853.
14. Motl RW, Pilutti L, Sandroff BM, Dlugonski D, Sosnoff JJ, Pula JH: **Accelerometry as a measure of walking behavior in multiple sclerosis**. *Acta Neurol Scand* 2013, **127**(6):384-390.
15. Autenrieth CS, Karrasch S, Heier M, Gorzelniak L, Ladwig KH, Peters A, Doring A: **Decline in gait performance detected by an electronic walkway system in 907 older adults of the population-based KORA-Age study**. *Gerontology* 2013, **59**(2):165-173.
16. McGough EL, Logsdon RG, Kelly VE, Teri L: **Functional mobility limitations and falls in assisted living residents with dementia: physical performance assessment and quantitative gait analysis**. *J Geriatr Phys Ther* 2013, **36**(2):78-86.
17. Paterson KL, Hill KD, Lythgo ND, Maschette W: **The reliability of spatiotemporal gait data for young and older women during continuous overground walking**. *Arch Phys Med Rehabil* 2008, **89**(12):2360-2365.
18. Lord S, Galna B, Verghese J, Coleman S, Burn D, Rochester L: **Independent domains of gait in older adults and associated motor and nonmotor attributes: validation of a factor analysis approach**. *J Gerontol A Biol Sci Med Sci* 2013, **68**(7):820-827.
19. Beauchet O, Freiberger E, Annweiler C, Kressig RW, Herrmann FR, Allali G: **Test-retest reliability of stride time variability while dual tasking in healthy and demented adults with frontotemporal degeneration**. *J Neuroeng Rehabil* 2011, **8**(1):37.
20. Beijer TR, Lord SR, Brodie MA: **Comparison of Handheld Video Camera and GAITRite(R) Measurement of Gait Impairment in People with Early Stage Parkinson's Disease: A Pilot Study**. *J Parkinsons Dis* 2013, **3**(2):199-203.
