# Supplementary material for: Comparison of programs for determining temporal-spatial gait variables from instrumented walkway data: PKmas versus GAITRite
Source: BMC Res Notes. 2014 Aug 18;7:542. doi: 10.1186/1756-0500-7-542 (PMC4150969; doi:10.1186/1756-0500-7-542)
Supplement: Supplementary file 2 — Additional file 2: B.pdf – Differences in calculations to derive variables between GAITRite® and PK mas® . (PDF 101 KB) [file 13104_2013_3088_MOESM2_ESM.pdf]

Additional file B

Differences in definitions and calculations between GAITRite® and PKMAS®. Information sourced from GAITRite® (http://www.gaitrite.com/downloads/WI-02-15_Technical_Reference_L.pdf) and Prokinetics (http://www.protokinetics.com/images/PKMAS_Gait_Measurement_Definitions_Short.pdf).

1. Base Width:

PKMAS® calculates Base Width ('stride width') as the perpendicular distance from heel reference point to the line connecting the heel reference points of the opposite side stride. PKMAS® uses a 'minimum volume ellipse' method to identify footfalls. The heel reference point is the intersection between the main axis of the ellipse and the hindmost end of the ellipse. In GAITRite® the 'HH base of support' measure is also the perpendicular distance from the heel reference point to the line connecting the heel reference points of the opposite side. However in GAITRite® the heel reference point is the centre of the heel section of the footfall trapezoid. This means that with an outward foot angle greater than zero degrees, the GAITRite® Base Width measure will be larger than the PKMAS® base width measure. The greater the amount of Foot Angle, the larger the difference between the two Base Width values.

GAITRite®

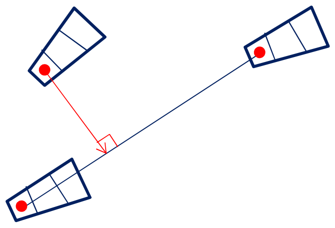

PKMAS®

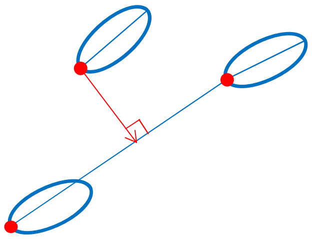

2. Step Length:

In brief, PKMAS® determines step length relative to the opposite stride, while GAITRite® determines step length relative to the walkway. Thus PKMAS® takes into account the instantaneous direction of progression while GAITRite® does not. In addition, the variables of Stride Length and Step Length also use the same method of locating the heel reference point as for Base Width which will also lead to small differences in the values. These small differences that do not noticeably affect the resulting values for Step Length if the person is walking reasonably straight, can still result in relatively larger differences in the standard deviation for Step length. If the direction of progression of the walk is not parallel to the mat, the values can differ substantially. As an example, the Step Length from a hip fracture participant (mean of all their Step Lengths) was calculated as 43.4cm with both GAITRite® and PKMAS®, but the standard deviation of the Step Length was 6.2cm and 4.2cm respectively.

GAITRite®

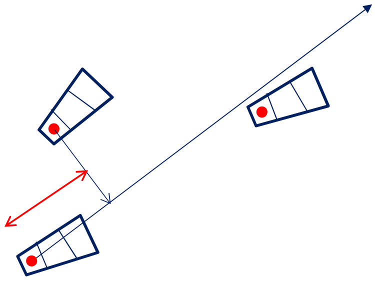

PKMAS®

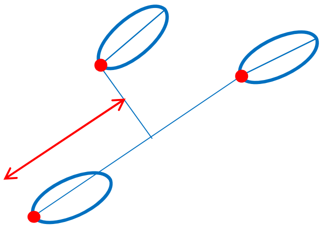

3. Foot Angle:

Foot angle is defined as the angle between the line of progression and the midline of the foot. The two programs have different methods of determining both these dimensions. In GAITRite® the midline of the foot is the line between the centre of the heel section of the footfall trapezoid and the centre of the forefoot section of the trapezoid. In PKmas® it is the major axis through the ellipse bounding the footprint. Differences in how the heel is weighted for each step will influence the location of the heel centre in the GAITRite® method more than in the PKmas® method. Differences in line of progression calculation will also contribute to differences in Foot Angle values. In GAITRite®, for Foot Angle calculation, the line of progression is determined from the footfalls by the opposite foot before and after the footfall of interest. However, PKmas® uses the footfall of interest plus the next footfall of the same foot to determine line of progression.

GAITRite®

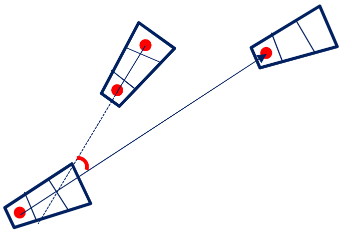

PKMAS®

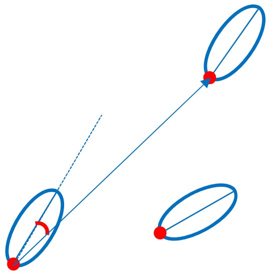

4. Temporal variables:

There is a slight difference in calculations for Stance and Swing Duration where PKMAS® Stance Duration is one sample time longer and Swing Duration is one sample time (0.125s) shorter than for GAITRite®. These differences appeared to make almost no difference to the ICCs but a small systematic difference is evident, where PKMAS® Stance Duration values tend to be higher (0.005s) and Swing Duration values are lower (0.003s). There is also a difference in the foot contact time determination that will affect all timing variables. PKMAS® is more sensitive to including the time the final sensor is still 'on' in the foot contact time, leading to a tendency for contact times to be longer in PKMAS®. This will lead to systematically longer Stance and Double Support Durations, and corresponding percentages of gait cycle, although these differences are clinically insignificant.
